# M2-like tumor-associated macrophages may promote tumor progression in malignant pleural mesothelioma

**DOI:** 10.1016/j.tranon.2025.102324

**Published:** 2025-02-20

**Authors:** Tetsuya Fukui, Ryota Sumitomo, Toshi Menju, Masashi Kobayashi, Hiroaki Sakai, Hiroshi Date

**Affiliations:** a Department of Thoracic Surgery, Graduate School of Medicine, Kyoto University, 54 Shogoin-Kawahara-cho, Sakyo-ku, Kyoto 606-8507, Japan; b Department of Thoracic Surgery, Kurashiki Central Hospital, Okayama 710-0052, Japan; c Department of Thoracic Surgery, Hyogo Prefectural Amagasaki General Medical Center, Hyogo 660-8550, Japan

**Keywords:** Tumor-associated macrophage, Mesothelioma, Progression, Histology, Tumor cell proliferation

## Abstract

**Introduction:**

Malignant pleural mesothelioma (MPM) is an aggressive malignancy with an unfavorable prognosis. Asbestos-activated macrophages may contribute to both oncogenesis and progression of MPM. This study aimed to clarify the biological and clinical significance of M2-like tumor-associated macrophages (TAMs) in MPM.

**Methods:**

This retrospective study included 101 MPM patients who were diagnosed and started treatment between 1998 and 2010. The distribution of M2-like TAMs in the intratumoral and peritumoral regions was evaluated by immunohistochemistry using CD163 staining. Tumor proliferation was evaluated by Ki-67 staining.

**Results:**

Intratumoral M2-like TAM density was significantly correlated with the pretreatment C-reactive protein level (*r* = 0.283, *P* = 0.004) and Ki-67 proliferation index (*r* = 0.498, *P* < 0.001). Peritumoral M2-like TAM density was also significantly correlated with the pretreatment C-reactive protein level (*r* = 0.255, *P* = 0.010) and Ki-67 proliferation index (*r* = 0.435, *P* < 0.001). Additionally, intratumoral M2-like TAM density was associated with histological subtype (*P* < 0.001), with higher densities observed in sarcomatoid tumors compared to epithelioid tumors. The overall survival rate was significantly worse in the intratumoral and peritumoral M2-like TAM-high groups (*P* = 0.044 and *P* = 0.046, respectively), particularly in patients with advanced-stage MPM. Multivariable analysis identified peritumoral M2-like TAM status (hazard ratio = 1.700, 95 % confidence interval: 1.034–2.796, *P* = 0.037), clinical stage, and histology as significant prognostic factors for overall survival.

**Conclusions:**

During MPM progression, M2-like TAMs may induce tumor cell proliferation and aggressiveness, contributing to the poor prognosis in MPM patients.

## Highlights

• Malignant pleural mesothelioma (MPM) is an aggressive disease with a poor prognosis.
• The significance of M2-like tumor-associated macrophages (TAMs) was assessed in MPMs.
• High M2-like TAM density linked to higher C-reactive protein and Ki-67 index.
• In sarcomatoid tumors, M2-like TAMs were more abundant than in epithelioid tumors.
• High M2-like TAM density was associated with worse overall survival in MPM patients.

## Introduction

Malignant pleural mesothelioma (MPM) remains a highly invasive malignancy with an unfavorable prognosis, ranging from 8 to 20 months [1,2]. Epidemiological and experimental studies have demonstrated that MPM is an occupational disease primarily caused by asbestos exposure [3,4]. Normal phagocytic processes are unable to break down inhaled asbestos fibers, which remain in the pleural cavity and continuously activate macrophages. When macrophages phagocytose asbestos fibers, it triggers the formation of the inflammasome complex and enhances the release of IL-1β [5]. IL-1β plays a key role in the oncogenesis of asbestos-induced mesothelioma [6]. Recent experimental studies have reported that MPM cells induce macrophages towards an M2-like phenotype [7]. In turn, cytokines including transforming growth factor β and IL-1β secreted from M2-like TAMs contribute to promoting the malignant potential of MPM cells [8,9]. Thus, macrophages not only contribute to the oncogenesis of MPM but might also play a significant role in its progression.

Therefore, this comprehensive study aimed to clarify the biological and clinical significance of M2-like TAMs in MPM.

## Materials and methods

### Patients

The study included consecutive patients diagnosed with MPM and treated at Kyoto University Hospital or Hyogo Prefectural Amagasaki General Medical Center between January 1998 and December 2010. The Ethics Committee at Kyoto University Hospital (approval no. R1706) and Hyogo Prefectural Amagasaki General Medical Center (approval no. 6–154) approved the study, and each patient provided written informed consent. All tumors were staged using the International Mesothelioma Interest Group's system, and the medical records, including detailed histopathological reports, were carefully documented. Follow-up data were available up to November 2023.

### Immunohistochemistry

Immunohistochemical analyses were conducted to investigate the distribution of M2-like tumor-associated macrophages (TAMs) in the intratumoral and peritumoral regions using CD163 staining and to evaluate the proliferation rate of tumors using the Ki-67 proliferation index. The Ventana BenchMark GX system (Ventana Medical Systems, Tucson, AZ, USA) or manual methods were employed for these studies. The following antibodies were applied: mouse monoclonal anti-human CD163 (#760–4437; prediluted; Ventana Medical Systems) and mouse monoclonal anti-human Ki-67 antibody (#M7240; diluted at 1:40; Agilent, Santa Clara, CA, US). Slides were prepared with 4-mm sections of formalin-fixed paraffin-embedded tissue and coated with poly-l-lysine. After deparaffinization and rehydration, the antigen retrieval process was initiated using Cell Conditioner 1 (Ventana Medical Systems) for 32 min at 100°C against CD163 or microwave in 10 µmol/L citrate buffer solution at pH 6.0 for 10 min against Ki-67. Subsequently, the sections were incubated with specific primary antibodies: CD163 for 16 min at 37°C and Ki-67 overnight at room temperature. Next, the sections were incubated with OptiView HQ Linker (Ventana Medical Systems) for 8 min at 37°C and OptiView HRP Multimer (Ventana Medical Systems) for 8 min at 37°C against CD163 or incubated with a biotinylated secondary antibody for 1 h, followed by incubation with the avidin-peroxidase complex for 1 h against Ki-67. The sections were then visualized with 3,3′-diaminobenzidine tetrahydrochloride (Dojindo Laboratories, Kumamoto, Japan), and lastly, counterstaining was performed with Mayer's hematoxylin.

Two investigators (TF and RS) assessed the immunohistochemical expression of four different fields, each containing at least 200 cells, while being blinded to the patients’ clinical data. Discrepancies between their assessments were resolved through joint review until a consensus was achieved. The number of CD163-positive cells and Ki-67-positive cells was counted manually. The intratumoral region was defined as a field within the tumor, or, if not feasible, as a field containing at least 70 % tumor tissue. The peritumoral region was defined as a field adjacent to the tumor where >70 % of the tissue consists of stroma. The intratumoral and peritumoral densities of CD163-positive macrophages were defined as intratumoral and peritumoral M2-like TAM densities and determined as the number of cells per mm^2^. The Ki-67 proliferation index was determined by scoring the percentage of carcinoma cells with positive staining for Ki-67 in a given specimen.

### Statistical analysis

For the analysis of continuous variables, a Student's *t*-test, one-way ANOVA followed by Dunnett's post hoc test, or Pearson's correlation coefficient was used. Categorical variables were assessed using a χ^2^ test. To determine the cut-off value for M2-like TAM density, we employed the minimum p-value approach. Specifically, we used continuous data of pretreatment C-reactive protein (CRP) level to dichotomize the study population based on various M2-like TAM density cutoff values. For each cutoff value, we conducted a *t*-test between the two groups, and the cut-off value yielding the most significant p-value was selected as the optimal cut-off. Overall survival (OS) was defined as the duration from the start of treatment to the date of death from any cause. The probability of OS over time was assessed using the Kaplan-Meier method, and differences in survival curves were compared using the log-rank test. The Cox regression model was employed for univariable and multivariable analyses to examine the influence on survival. Statistical analyses were conducted with SPSS 23.0 for Windows (IBM Corp., Armonk, NY, USA). Two-tailed statistical analyses were performed for all P-values, and a P-value <0.05 was deemed statistically significant.

## Results

### Patients

A total of 106 MPM patients were diagnosed and began treatment at Kyoto University Hospital or Hyogo Prefectural Amagasaki General Medical Center between January 1998 and December 2010. Five patients were excluded from the study due to incomplete pretreatment CRP level data, resulting in a total of 101 patients included in the analysis. The characteristics of these patients are presented in Table 1. Among these, there were 57 epithelioid tumors, 19 biphasic tumors, and 25 sarcomatoid tumors. Twenty-eight cases underwent surgical treatment, and all of them were treated with extrapleural pneumonectomy. Sixty-five patients received chemotherapy treatments: 31 on the cisplatin-gemcitabine regimen, 29 on cisplatin-pemetrexed, and 5 on other regimens. Twenty-three patients received radiotherapy.

**Table 1:** Clinicopathological characteristics among 101 MPM patients.

Variables		n
Age	≤ 65	52
	> 65	49
Gender	Female	28
	Male	73
Smoking history	Yes	62
	No	39
Asbestos exposure	Yes	43
	No	58
Clinical stage	I	13
	II	18
	III	36
	IV	34
Histology	Epithelioid	57
	Biphasic	19
	Sarcomatoid	25
Surgical resection	Yes	28
	No	73
Chemotherapy	Yes	65
	No	36
Radiotherapy	Yes	23
	No	78
Total number		101

MPM, malignant pleural mesothelioma.

### Distribution of M2-like TAMs in MPM

Intratumoral M2-like TAM density exhibited substantial variation among the 101 MPM specimens, with a mean ± standard deviation (SD) of 660.8 ± 565.9 cells/mm^2^ (Supplementary Fig. 1A, C, Table 2). The optimal cut-off value was determined to be 540.7. Samples were classified as intratumoral M2-like TAM-high if their intratumoral M2-like TAM density exceeded 540.7.

**Table 2:** Distributions of intratumoral and peritumoral M2-like TAM density among 101 MPM patients according to clinicopathological characteristics.

			Intratumoral M2-like TAM density		Peritumoral M2-like TAM density		Intratumoral M2-like TAM status			Peritumoral M2-like TAM status		
		n	mean ± SD (cells per mm^2^)	P	mean ± SD (cells per mm^2^)	P	Low	High	P	Low	High	P
Age	≤ 65	52	555.5 ± 463.1	0.279^a^	214.0 ± 161.0	0.633^a^	27	25	0.232^c^	28	24	1.000^c^
	> 65	49	666.6 ± 560.6		232.7 ± 228.0		19	30		27	22	
Gender	Female	28	536.6 ± 492.0	0.380^a^	199.1 ± 186.6	0.448^a^	14	14	0.658^c^	14	14	0.658^c^
	Male	73	637.4 ± 521.6		232.3 ± 199.5		32	41		41	32	
Smoking history	Yes	62	602.1 ± 559.2	0.857^a^	223.2 ± 211.9	0.995^a^	30	32	0.541^c^	36	26	0.414^c^
	No	39	621.1 ± 436.7		222.9 ± 169.1		16	23		19	20	
Asbestos exposure	Yes	43	633.3 ± 587.1	0.690^a^	228.1 ± 218.4	0.825^a^	20	23	1.000^c^	25	18	0.550^c^
	No	58	591.7 ± 455.3		219.4 ± 178.8		26	32		30	28	
Clinical stage	I	13	595.3 ± 619.0	0.439^b^	215.3 ± 110.8	0.467^b^	6	7	0.043c*	6	7	0.561^c^
	II	18	784.6 ± 385.4		264.0 ± 183.3		3	15		9	9	
	III	36	543.8 ± 516.9		185.1 ± 174.8		20	16		23	13	
	IV	34	591.6 ± 526.2		244.6 ± 242.6		17	17		17	17	
Histology	Epithelioid	57	422.3 ± 410.4	< 0.001b*	206.7 ± 189.9	0.213^b^	36	21	< 0.001c*	33	24	0.522^c^
	Biphasic	19	712.5 ± 337.4		194.2 ± 160.5		3	16		11	8	
	Sarcomatoid	25	957.6 ± 633.3		282.4 ± 226.0		7	18		11	14	
Total number		101	660.8 ± 565.9		223.1 ± 195.6		46	55		55	46	

⁎ *P* < 0.05.

a P-value determined using a *t*-test.

b P-value determined using one-way ANOVA followed by a Dunnett test.

c P-value determined using c2 test. MPM, malignant pleural mesothelioma. TAM, tumor-associated macrophage.

Peritumoral M2-like TAM density ranged widely among the 101 MPM specimens, with a mean ± SD of 223.1 ± 195.6 cells/mm^2^ (Supplementary Fig. 1E, F, Table 2). The cut-off value was optimally set at 223.1, and samples were classified as peritumoral M2-like TAM-high if their peritumoral M2-like TAM density exceeded 223.1.

**Fig. 1:**
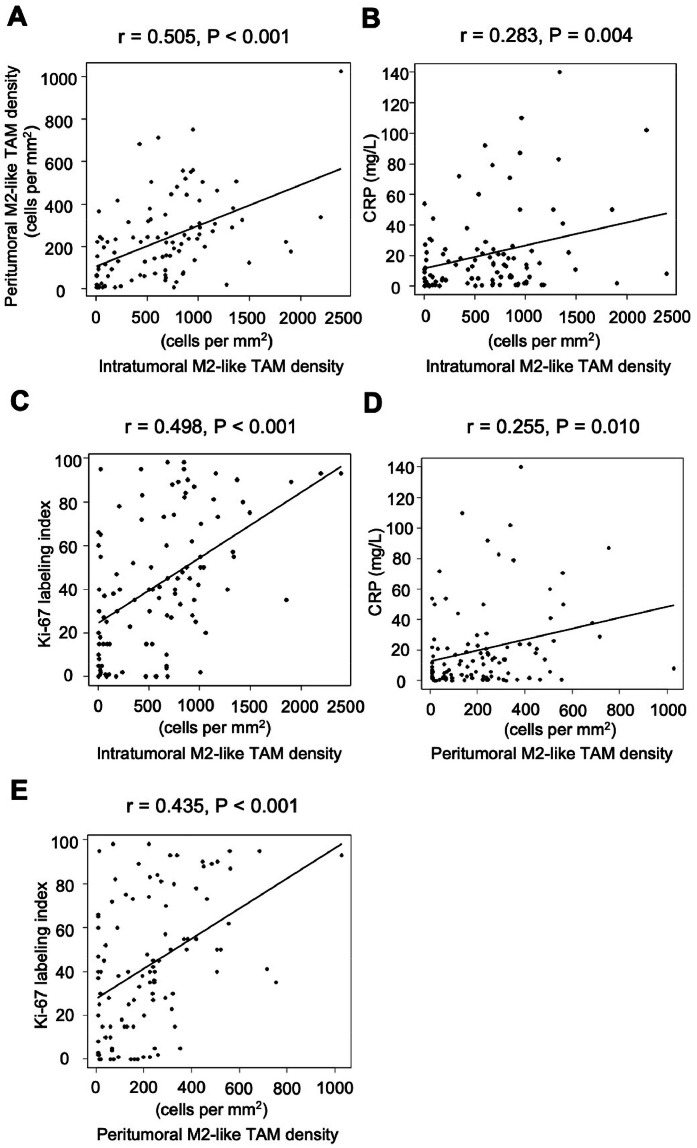
Correlation analysis. (A) Correlation between the intratumoral and peritumoral M2-like TAM densities. (B) Correlation between the intratumoral M2-like TAM density and pretreatment CRP level. (C) Correlation between the intratumoral M2-like TAM density and Ki-67 proliferation index. (D) Correlation between the peritumoral M2-like TAM density and pretreatment CRP level. (E) Correlation between the peritumoral M2-like TAM density and Ki-67 proliferation index. TAM, tumor-associated macrophage. CRP, C-reactive protein.

There was a significant correlation between the intratumoral and peritumoral M2-like TAM densities (*r* = 0.505, *P* < 0.001) (Fig. 1A).

### Systemic inflammation and tumor cell proliferation in MPM

**Fig. 2:**
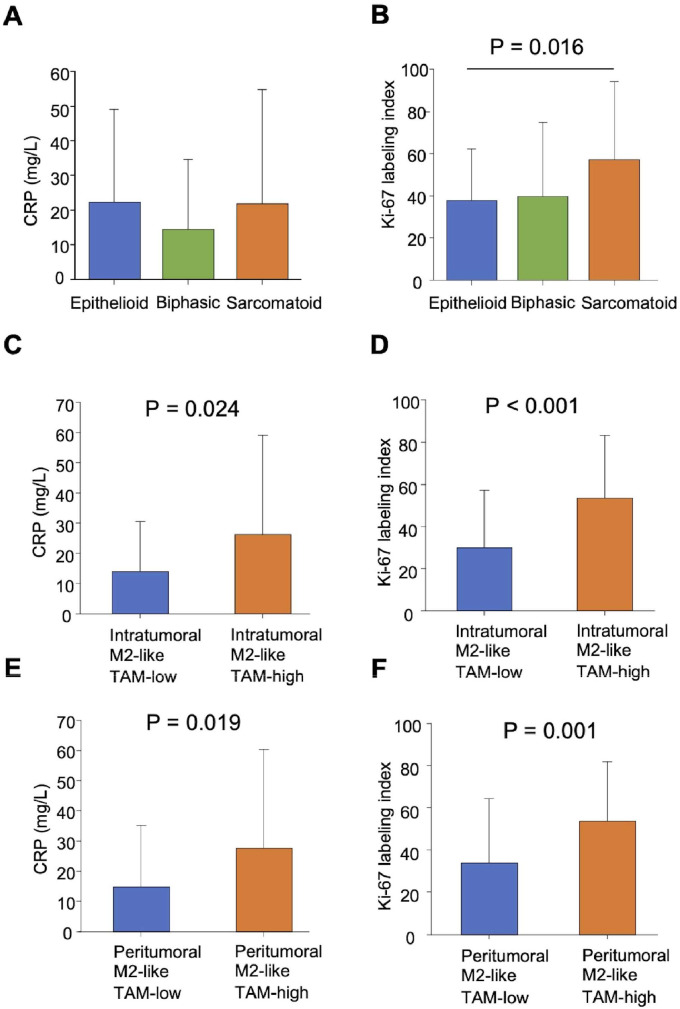
Biological significance of the M2-like TAMs in MPM. (A) Pretreatment CRP level according to histology. (B) Ki-67 proliferation index according to histology. (C) Pretreatment CRP level according to intratumoral M2-like TAM status. (D) Ki-67 proliferation index according to intratumoral M2-like TAM status. (E) Pretreatment CRP level according to peritumoral M2-like TAM status. (F) Ki-67 proliferation index according to peritumoral M2-like TAM status. TAM, tumor-associated macrophage. MPM, malignant pleural mesothelioma. CRP, C-reactive protein.

The mean ± SD of the pretreatment CRP level was 20.7 ± 27.3 mg/L. According to histology, no significant difference was observed between epithelioid, biphasic, and sarcomatoid tumors (Fig. 2A). The mean ± SD of the Ki-67 proliferation index in MPM tumors was 42.8 ± 30.8 % (Supplementary Fig. 1B, D). According to histology, the Ki-67 proliferation index was significantly higher in the sarcomatoid tumors compared to that in the epithelioid tumors (57.1 ± 37.0 % vs. 37.6 ± 24.4 %, *P* = 0.016) (Fig. 2B).

### The significance of intratumoral M2-like TAMs in MPM from biological and clinical perspectives

A weak correlation was found between M2-like TAM density and pretreatment CRP level (*r* = 0.283, *P* = 0.004) (Fig. 1B). The intratumoral M2-like TAM-high group exhibited a significantly higher pretreatment CRP level compared to the intratumoral M2-like TAM-low group (26.2 ± 33.0 vs. 14.0 ± 16.5 mg/L, *P* = 0.024) (Fig. 2C). Additionally, there was a significant correlation between intratumoral M2-like TAM density and the Ki-67 proliferation index (*r* = 0.498, *P* < 0.001) (Fig. 1C). The Ki-67 proliferation index was significantly elevated in the intratumoral M2-like TAM-high group compared to the intratumoral M2-like TAM-low group (53.6 ± 29.7 % vs. 30.0 ± 27.2 %, *P* < 0.001) (Fig. 2D).

From a clinical viewpoint, there was a significant association between intratumoral M2-like TAM density and histology (*P* < 0.001) (Table 2). Moreover, the status of intratumoral M2-like TAMs was significantly related to both clinical stage (*P* = 0.043) and histology (*P* < 0.001).

### The significance of peritumoral M2-like TAMs in MPM from biological and clinical perspectives

M2-like TAM density showed a weak correlation with pretreatment CRP level (*r* = 0.255, *P* = 0.010) (Fig. 1D). The peritumoral M2-like TAM-high group had significantly higher pretreatment CRP levels compared to the peritumoral M2-like TAM-low group (27.6 ± 32.7 vs. 14.9 ± 20.4 mg/L, *P* = 0.019) (Fig. 2E). Additionally, peritumoral M2-like TAM density showed a significant correlation with the Ki-67 proliferation index (*r* = 0.435, *P* < 0.001) (Fig. 1E). The peritumoral M2-like TAM-high group had a significantly higher Ki-67 proliferation index compared to the peritumoral M2-like TAM-low group (53.5 ± 28.1 % vs. 33.9 ± 30.4 %, *P* = 0.001) (Fig. 2F).

From a clinical standpoint, neither peritumoral M2-like TAM density nor status showed significant differences with clinicopathological factors (Table 2).

### The impact of intratumoral M2-like TAM status on the overall survival of MPM patients

**Fig. 3:**
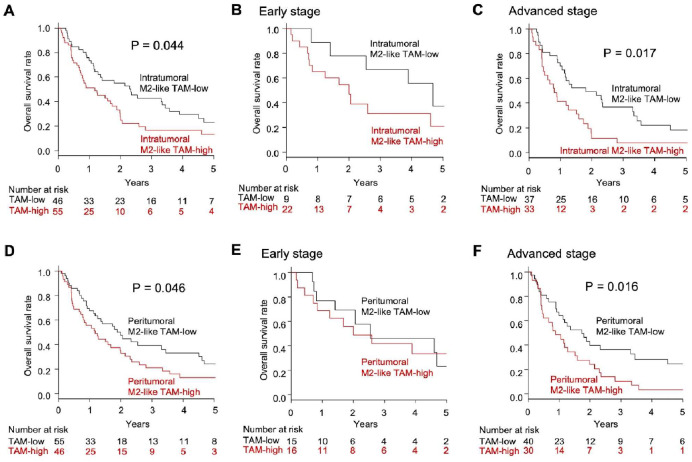
Overall survival. (A) Overall survival among 101 MPM patients according to intratumoral M2-like TAM status. (B) Overall survival among 31 patients with early-stage MPM according to intratumoral M2-like TAM status (C) Overall survival among 70 patients with advanced-stage MPM according to intratumoral M2-like TAM status. (D) Overall survival among 101 MPM patients according to peritumoral M2-like TAM status. (E) Overall survival among 31 patients with early-stage MPM according to peritumoral M2-like TAM status (F) Overall survival among 70 patients with advanced-stage MPM according to peritumoral M2-like TAM status. MPM, malignant pleural mesothelioma. TAM, tumor-associated macrophage.

The median OS was 15.0 months in the present study. The OS rate was significantly reduced in the intratumoral M2-like TAM-high group compared to the intratumoral M2-like TAM-low group (13.3 % vs. 23.2 % in 5-year OS, *P* = 0.044) (Fig. 3A). Among early-stage MPM patients, OS rates did not differ significantly based on intratumoral M2-like TAM status (Fig. 3B). However, among advanced-stage MPM patients, the intratumoral M2-like TAM-high group had a significantly lower OS rate compared to the intratumoral M2-like TAM-low group (7.8 % vs. 18.4 % in 5-year OS, *P* = 0.017) (Fig. 3C).

In the univariable analysis, the Cox regression model showed that intratumoral M2-like TAM status (hazard ratio [HR] = 1.606, 95 % confidence interval [CI]: 1.009–2.557, *P* = 0.046) significantly predicted OS in MPM patients.

### The impact of peritumoral M2-like TAM status on the overall survival of MPM patients

The OS rate was significantly reduced in the peritumoral M2-like TAM-high group compared to the peritumoral M2-like TAM-low group (13.2 % vs. 24.1 % in 5-year OS, *P* = 0.046) (Fig. 3D). Among patients with early-stage MPM, there was no significant difference in OS rates based on peritumoral M2-like TAM status (Fig. 3E). Conversely, among patients with advanced-stage MPM, the OS rate was significantly decreased in the peritumoral M2-like TAM-high group compared to the peritumoral M2-like TAM-low group (3.5 % vs. 24.4 % in 5-year OS, *P* = 0.016) (Fig. 3F).

In the univariable analysis, the Cox regression model showed that peritumoral M2-like TAM status (HR = 1.590, 95 % CI: 1.004–2.517, *P* = 0.048) significantly predicted OS in MPM patients.

### Multivariable analysis

Univariable Cox regression analysis identified clinical stage, histology, surgical resection, intratumoral M2-like TAM status, and peritumoral M2-like TAM status as prognostic factors for OS (Table 3). Multivariable analysis of these significant univariable factors showed that peritumoral M2-like TAM status (HR = 1.700, 95 % CI: 1.034–2.796, *P* = 0.037), clinical stage (HR = 2.028, 95 % CI: 1.177–3.493, *P* = 0.011), and histology (HR = 1.690, 95 % CI: 1.029–2.777, *P* = 0.038) were significant prognostic factors for OS.

**Table 3:** Univariable and multivariable Cox regression analyses for overall survival among 101 MPM patients.

	Univariable		Multivariable	
Variables	HR (95 % CI)	P	HR (95 % CI)	P
Age (> 65)	1.266 (0.797–2.012)	0.318		
Gender (Male)	0.776 (0.469–1.286)	0.326		
Smoking history (Yes)	0.827 (0.514–1.329)	0.431		
Asbestos exposure (Yes)	0.745 (0.466–1.192)	0.219		
Clinical stage (III,IV)	1.713 (1.020–2.877)	0.042*	2.028 (1.177–3.493)	0.011*
Histology (Non-epithelioid)	1.957 (1.232–3.108)	0.004*	1.690 (1.029–2.777)	0.038*
Surgical resection (Yes)	0.486 (0.284–0.831)	0.008*	0.582 (0.334–1.014)	0.056
Chemotherapy (Yes)	1.312 (0.788–2.185)	0.296		
Intratumoral M2-like TAM status (High)	1.606 (1.009–2.557)	0.046*	1.404 (0.839–2.349)	0.197
Peritumoral M2-like TAM status (High)	1.590 (1.004–2.517)	0.048*	1.700 (1.034–2.796)	0.037*

⁎ *P* < 0.05. MPM, malignant pleural mesothelioma. TAM, tumor-associated macrophage. HR, hazard ratio. CI, confidence interval.

## Discussion

In the present study, both intratumoral and peritumoral M2-like TAM density showed a correlation with the pretreatment CRP level of MPM patients. CRP is a well-established systemic inflammatory marker that reflects the presence and intensity of inflammation in the body. This finding is consistent with the mechanism that chronic inflammation activates macrophages.

Furthermore, immunohistochemical analyses demonstrated that intratumoral and peritumoral M2-like TAM density showed a correlation with the Ki-67 proliferation index in this study. An experimental study reported that IL-1β secreted by M2-like TAMs induces the expression of CD26 in MPM cells, which contributes to the enhancement of the tumor's self-renewal capacity and sphere formation ability [9]. A study using a mouse model of renal clear cell carcinoma demonstrated that TAM depletion suppresses tumor growth and enhances natural killer cell activity [10]. In addition, Wu et al. have recently reported that reducing M2-like TAMs in malignant mesothelioma, via selective deletion of the Dicer1 gene in myeloid cells, led to the normalization of the tumor microenvironment and tumor rejection in a mouse model [11]. These findings highlight the critical role of M2-like TAMs in tumor progression. In contrast, a previous study using 93 MPM samples showed no correlation between intratumoral and peritumoral CD68-positive cells and Ki-67 proliferation index in MPM cells [12]. This observation indicates that the immunostaining for CD163, rather than CD68, may have revealed the correlation with the Ki-67 proliferation index [13]. The present study is the first to show that intratumoral and peritumoral M2-like TAM density is related to tumor cell proliferation in MPM.

Intratumoral M2-like TAM density had an association with histology in the present study. The spatial transcriptomic study by Torricelli et al. recently demonstrated that M2-like TAMs, marked by CD163 and MRC1, are predominant in the sarcomatoid regions of biphasic MPM tumors [8]. Furthermore, the enrichment of M2-like TAMs correlates with increased epithelial-to-mesenchymal transition (EMT) markers, suggesting that M2-TAMs promote the transition from epithelial to mesenchymal phenotypes [8,14]. M2-like TAMs contribute to the tumor microenvironment by promoting pathways associated with tumor progression, including EMT [11]. The mechanism by which M2-like TAMs induce EMT involves the secretion of various soluble factors, including IL-1β, transforming growth factor β, and IL-8, which are potent inducers of EMT and contribute to tumor cell invasion and migration [15]. These cytokines activate signaling pathways such as SMAD, STAT3, and NF-κB in tumor cells, leading to the downregulation of epithelial markers like E-cadherin and the upregulation of mesenchymal markers such as vimentin and N-cadherin [[14], [15], [16]]. These findings support the relationship between M2-like TAMs and histology observed in the present study.

Peritumoral M2-like-TAM status was a prognostic factor in multivariate Cox regression analysis in the present study. In univariate analysis, both intratumoral M2-like TAM status and peritumoral M2-like TAM status were significant predictors of OS. However, in multivariable analysis, only peritumoral M2-like TAM status remained a significant predictor. M2-like TAMs play important biological functions in tumor progression including vascularization, intravasation, and extravasation, establishing pre-metastatic niches [17]. In hepatocellular carcinoma samples, EMT cells were mainly found at the edge of tumor nests, while TAMs were primarily located at the border between tumor nests and stroma [15]. M2-like TAMs also secrete matrix metalloproteinases directly and indirectly through the tumor microenvironment, which degrade the extracellular matrix, a substrate as well as a barrier for tumor cell migration. Additionally, M2-like TAMs are reported to secrete chitinase 3-like protein 1, which upregulates matrix metalloproteinases expression, enhancing the invasiveness of gastric and breast cancer cells [18]. Peritumoral M2-like TAMs may be more closely associated with tumor progression and prognosis than intratumoral M2-like TAMs. Therefore, peritumoral M2-like TAMs might serve as a biomarker for therapeutic strategies for MPM. M2-like TAM was recently reported to be a promising biomarker for immunotherapy response in breast cancer [19]. Moreover, TAMs represent a potential therapeutic target in MPM. Given the pivotal role of M2-like macrophages in tumor progression, strategies aimed at reprogramming TAMs into a tumor-suppressing phenotype may offer a promising avenue for novel therapies [11,17,[20], [21], [22]]. Recently, toll-like receptor 7/8 agonists have attracted attention as therapeutic agents capable of re-educating M2-like TAMs into an M1-like phenotype [22,23].

This study has several limitations. First, the present study is retrospective and has a relatively small sample size. Second, the extended duration of the study period may have influenced changes in treatment strategies.

In conclusion, the present study showed that M2-like TAMs might promote tumor progression in MPM. TAMs have shown potential as a novel therapeutic target for MPM. Further studies are needed to develop effective treatment approaches.

## Funding

This work was supported by the Fujiwara Memorial Foundation grant for young researcher.

## CRediT authorship contribution statement

**Tetsuya Fukui:** Writing – original draft, Visualization, Methodology, Investigation, Formal analysis. **Ryota Sumitomo:** Writing – original draft, Visualization, Validation, Resources, Methodology, Investigation, Formal analysis, Conceptualization. **Toshi Menju:** Writing – review & editing, Resources, Methodology. **Masashi Kobayashi:** Writing – review & editing, Resources, Data curation. **Hiroaki Sakai:** Writing – review & editing, Resources, Data curation. **Hiroshi Date:** Writing – review & editing, Supervision.

## Declaration of competing interest

The authors declare that they have no known competing financial interests or personal relationships that could have appeared to influence the work reported in this paper.

## Appendix Supplementary materials

Image, application 1
