# Supplementary material for: M2-like tumor-associated macrophages may promote tumor progression in malignant pleural mesothelioma
Source: Transl Oncol. 2025 Feb 20;54:102324. doi: 10.1016/j.tranon.2025.102324 (PMC11889561; doi:10.1016/j.tranon.2025.102324)
Supplement: Supplementary file 1 [file mmc1.docx]

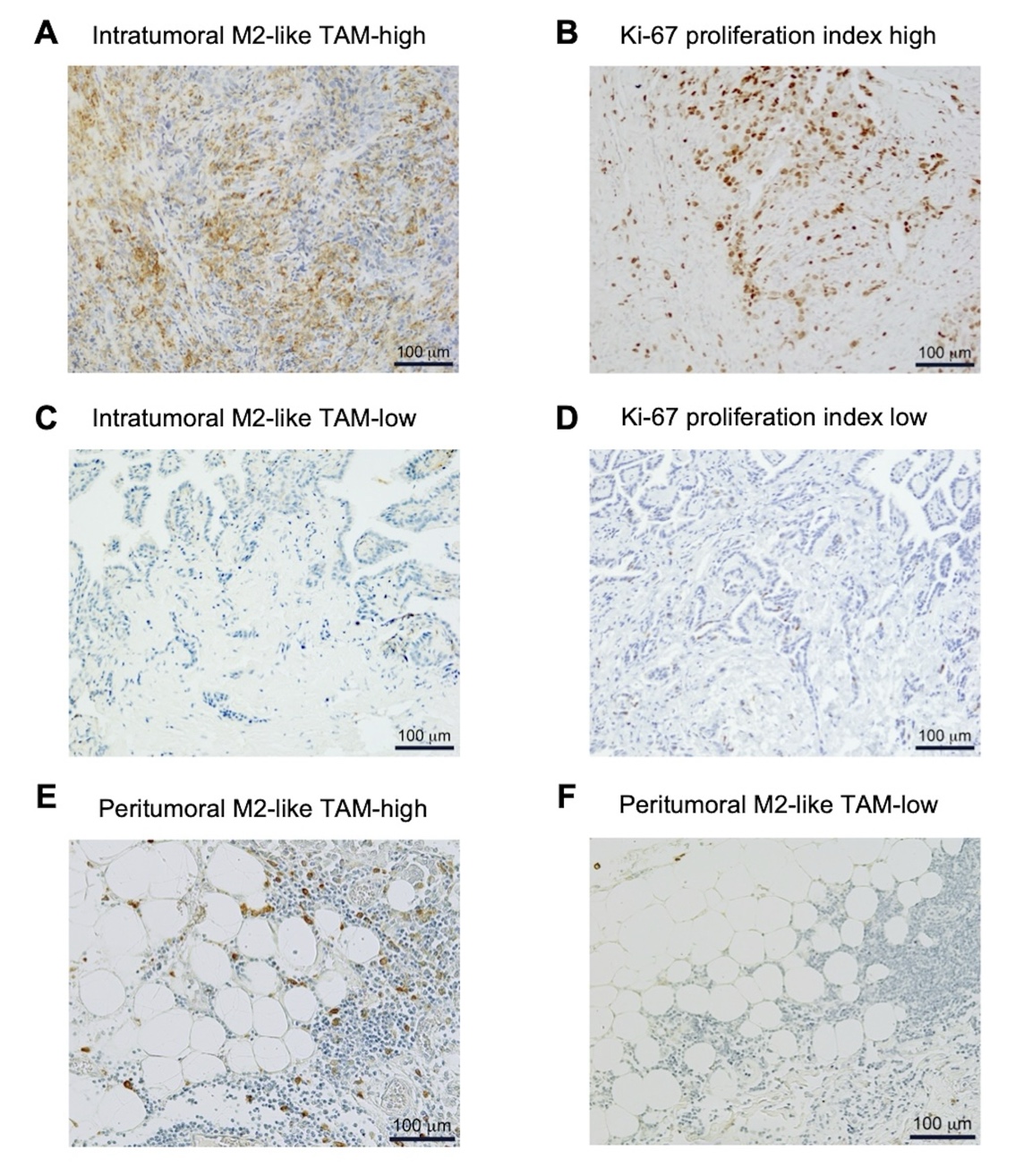


Supplementary Figure 1.

Immunostaining of MPM. An MPM tumor with high density of CD163-positive (M2-like) TAMs in the intratumoral region (A), and a high Ki-67 proliferation index (B). An MPM tumor with low density of CD163-positive (M2-like) TAMs in the intratumoral region (C), and a low Ki-67 proliferation index (D). An MPM tumor with high density of CD163-positive (M2-like) TAMs in the peritumoral region (E). An MPM tumor with low density of CD163-positive (M2-like) TAMs in the peritumoral region (F). Bar, 100 μm. MPM, malignant pleural mesothelioma. TAM, tumor-associated macrophage.
